# Between Shifting and Feedback Processing in the Wisconsin Card Sorting Test in Children with Developmental Language Disorder

**DOI:** 10.3390/brainsci13081128

**Published:** 2023-07-26

**Authors:** Kristina Giandomenico, Lauren S. Baron, Asiya Gul, Yael Arbel

**Affiliations:** MGH Institute of Health Professions, Boston, MA 02129, USA; lbaron@mghihp.edu (L.S.B.); agul@mghihp.edu (A.G.); yarbel@mghihp.edu (Y.A.)

**Keywords:** developmental language disorder, executive function, set shifting, feedback processing, event-related potentials, WCST

## Abstract

Children with developmental language disorder (DLD) demonstrate deficits in executive functioning; however, the specific components of executive functioning that are affected in this population are not well understood. This study evaluated set shifting and feedback processing in a Wisconsin Card Sorting Test (WCST) performed by 24 children with and without DLD. The behavioral results revealed poorer performance by the DLD group in measures of accuracy, proportion of correct rule shifts, perseverative errors on shift trials, and proportion of effective responses to feedback. Electrophysiological measures (event-related potentials, or ERPs) indicated different patterns of response to negative feedback that signaled the need for rule shifting, with the DLD group showing a trend toward processing shift cues as negative feedback. Group differences were found in the processing of the first and last positive feedback, with overall stronger responses to positive feedback by children with DLD. However, both groups showed a similar pattern of diminished attention to positive feedback when rule learning was established. Taken together, children with DLD demonstrated the inefficient processing of negative feedback in the context of rule-shifting and difficulty in establishing and maintaining a rule.

## 1. Introduction

Developmental language disorder (DLD) is a neurodevelopmental condition that involves significant difficulty with learning, understanding, and using spoken language and is not associated with a known biomedical etiology [1,2]. Though there is a persisting lack of consensus on the exact diagnostic criteria of the disorder, DLD, or “language disorder of unknown origin,” has an estimated prevalence rate of more than 7% [1]. 

While DLD is often conceptualized as a domain-specific deficit, converging evidence has demonstrated that children with DLD may also demonstrate weaknesses in executive function relative to their peers [3,4,5]. As both language and executive function (EF) skills are developed through childhood, the nature of a bidirectional relationship between these skills is an important consideration; while the development of EF may be supported by the development of language, EF abilities may also be essential to language learning. A better understanding of EF strengths and weaknesses may influence how children at risk of language delay are identified and may also inform how effective interventions for these children can be structured and provided [6]. To learn more about the interplay between components of EF and language development in children with DLD, it is necessary to examine EF in a way that captures cognitive processes central to learning and is also conducive to differentiating between those processes. The notion of a general deficit in executive functioning in children with DLD has been proposed based on their relatively poor performance on typical tests of EF when compared to age-matched peers [3]. However, most EF tasks are complex and recruit multiple skills. The Wisconsin Card Sorting Test (WCST) is a commonly used assessment of executive function that has been found to be most strongly correlated with the EF component of shifting [7], also referred to as attention shifting or cognitive flexibility. Though performance was found to be predicted by shifting ability, the WCST is a complex task that involves skills of inhibition, problem-solving, feedback processing, abstract thinking, concept formation, and working memory [7,8]. Miyake et al. [7] made the argument that in complex executive tasks such as the WCST, factors other than those presumed to be primary to the task may be contributing substantially to performance and that this could be reflected in different strategies or approaches to the task employed by an individual. In studies that have aimed to investigate general executive functioning in children with DLD using the WCST, some have found group differences suggesting poorer performance by children with DLD as compared to typically developing children [9,10], while another study did not reveal significant differences between groups [11]. However, these studies primarily used total errors and perseveration errors as indices of performance on the task. Using more detailed performance analysis as well as investigating electrophysiological (event-related potentials or ERPs) data may serve to better elucidate the nature of performance patterns that may have led to these equivocal findings. The present study was designed to provide an in-depth behavioral and electrophysiological evaluation of the areas of weakness associated with the performance of children with DLD on the WCST.

### 1.1. Literature Review

#### 1.1.1. Unity and Diversity of EFs in Children with DLD

According to the unity and diversity theory of EF, there are three separable but correlated components of executive function: updating (i.e., working memory), inhibition, and shifting [7]. Updating entails the processes of monitoring and revising information and holding relevant information in a state that is accessible for carrying out the task at hand [7]. Inhibition refers to the ability to override the tendency to produce a dominant, pre-potent response [7]. Shifting is suggested to represent the ability to switch between multiple tasks and mental sets [7]. Due to the multifaceted nature of most tasks used to assess executive functioning, a poor outcome on any single task may not denote impaired EF overall but rather implicate a deficiency in a more specific component of EF. Conversely, it is misleading to assume that poor performance on a single task that is assumed to be strongly associated with one specific EF component is driven by that one component and not by a common ability underlying multiple EFs [7,8]. 

Previous studies have aligned in evidence that DLD is linked to deficits in verbal working memory, nonverbal inhibition, and both verbal and nonverbal sustained attention—yet research into other executive skills has resulted in more conflicting findings [3,4]. One example is shifting, also referred to as attention shifting or cognitive flexibility, which represents a skill set key to many learning tasks: developing and maintaining a representation of a rule in working memory and then switching to a new rule while inhibiting a previously formed rule [7,12]. In an investigation of math and reading skills, a meta-analysis by Yeniad et al. [12] concluded that a significant association exists between shifting abilities and both math and reading abilities in children. Given considerable variability in the results of studies of attentional shifting in children with DLD, Aljahlan and Spaulding [13] conducted a meta-analysis to investigate measures of shifting. The study reported that children with DLD exhibit deficits in set-shifting tasks (i.e., those requiring the modification of behavior after a period of repetitions, with infrequent rule changes) but do not differ from typically developing peers in alternating tasks (i.e., those requiring responses in a consistent pattern to an alternating set of rules). However, overall, there has been a paucity of research investigating the shifting component of EF in children with DLD and a limited understanding of the nature of a potential relationship [3]. 

#### 1.1.2. Wisconsin Card Sorting Test as a Test of EF

**Figure 1:**
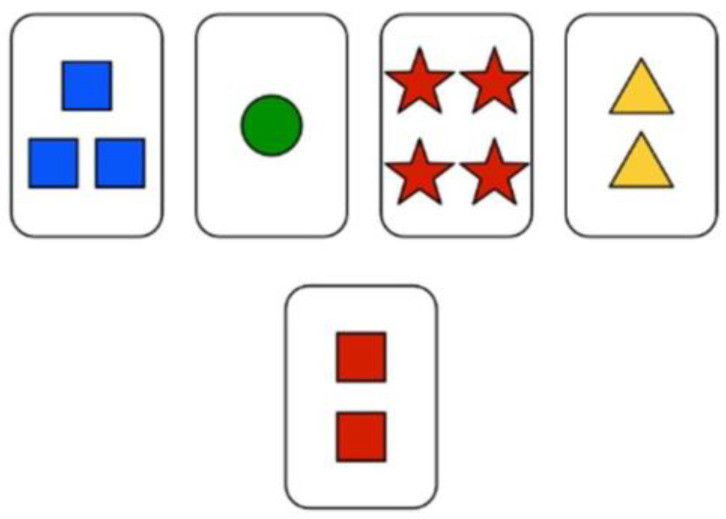
Example of Wisconsin Card Sorting Test (WCST) stimulus with key cards (**top row**) and a choice card (**bottom row**).

The WCST task has traditionally been employed to evaluate frontal lobe functioning based on the understanding of its dependence on higher-order cognitive processing [14]. In the task, the participant is required to match a series of choice cards to one of four fixed key cards based on three possible categories: the color, shape, or number of the figures on each card. An example of the card images used in this study’s version of the task is presented in Figure 1. Initially, the rule must be guessed by trial and error, based on feedback provided on whether the sorting decision was right or wrong. After a certain number of trials in accordance with one matching rule, the rule changes without notice to the participant—the change must be inferred based on new negative feedback elicited from a previously correct sorting principle. 

In the context of the WCST, performance can be evaluated based on how quickly and efficiently the participant finds the card sorting rule, as well as how well they are able to maintain it. Traditional scoring of the WCST considers the total number of errors, number of perseverative errors (i.e., continuing to respond based on the previous rule after a rule change has been indicated), and number of sets abiding by a single rule successfully completed. During the task, at the set-shift stage, an ideal participant would respond to the initial negative feedback indicating a rule change by selecting a card based on one of the two remaining rules (with a 50% chance of guessing the correct rule) and then either maintain or update their next response depending on the valence of the second feedback received. This ideal participant would therefore be able to infer the new rule by the second trial following the initial negative feedback, hold that new rule in mind to sort the next series of cards, and make no further errors until the next unexpected rule change. Errors made over the course of the task may reflect a failure to successfully shift cognitive sets to the new rule but may also reflect a failure to maintain the new rule due to distraction from competing stimuli (i.e., the multiple features of the card that must be considered) [15]. Barceló and Knight [15] associated set-switching ability with the measure of perseverative errors or the failure to switch to a new rule after negative feedback. They quantified set maintenance based on a measure of distraction errors, which are random losses of the sorting rule. 

While failure to change to a new sorting rule could indicate a lack of cognitive flexibility to adjust to the new demands of the task, it could also reflect ineffective utilization of the feedback signaling the need to shift. In the WCST, feedback on task performance has both valence and informative value—it relates to whether current behavior is right or wrong, and it provides information that can be used to adjust behavior to improve on subsequent trials. Therefore, beyond shifting ability, performance on the WCST is also linked to the efficient use of feedback. Successful completion of the WCST hinges on the ability to use feedback to initiate the shift by applying a different rule as well as to optimize behavior on a given rule within each set [16]. The ability to effectively interpret and utilize external feedback is a component of executive control [17]. Arbel et al. [18] examined the effect of feedback on learning in children with DLD, finding that both behavioral and electrophysiological data suggest an impairment in feedback processing in this population. In the WCST, both set-shifting and set-maintenance hinge on the processing of negative feedback as well as positive feedback, making feedback processing ability a key factor to take into consideration when examining the performance of children with DLD on this task.

Given the evidence that performance on the WCST may implicate weaknesses in different types of cognitive processes, analyses of the number, types, and patterns of errors on the task alone may not provide a full picture of the deficits that may lead to those errors. To better understand the processes involved in the set-shifting stage of the WCST, a closer look at the neurophysiological correlates elicited during task performance may have the potential to separate and pinpoint such suspected differences in processing.

#### 1.1.3. Event-Related Potentials and the WCST

Previous studies of WCST performance have highlighted ERPs related to feedback processing (feedback-related negativity; FRN) as well as those reflective of context updating and shift detection (P3a, P3b) as indicators of key processes involved in the completion of the task [16,19]. 

The P300 signal is a positive-going ERP component indicative of the process of shifting attention to update behaviors to new task demands and the revision of an established representation of the environment [16,20]. It is suggested to indicate the activation of a response system, which is represented by two distinct aspects—the frontal P3a, reflecting processes of attention switching, and the posterior P3b, reflecting processes of context updating [19]. Using a computerized WCST paradigm, Barceló [19] compared the first negative feedback presentation, the second negative feedback presentation, and subsequent “stay” feedback cues. The study found that the first two negative feedback events indicating a shift to a new task rule elicited a P3a response, which was diminished in response to the first feedback instructing participants to stay with the same rule. Further, the amplitude of the P3a was sharply reduced in response to the second stay cue as compared to the first stay cue. These findings supported the view of the P3a response as a reflection of the executive control of set-shifting [19]. The P3b activity observed by Barceló [19] was found to be sensitive to the number of rules held in memory, such that it decreased in amplitude from the first shift cue to the second shift cue and from the first stay cue to the second stay cue but did not differ between the second shift and the first stay cues. 

Cunillera et al. [16] used a modified version of the WCST in which participants were given positive and negative feedback cues, indicating the accuracy of the previous sort, as well as switch and stay cues, instructing participants to use a different sorting rule or repeat the rule that applied on the previous trial. Their results revealed a P300 response evoked by switch cues but not stay cues, along with a similar P300 in response to the first positive feedback signal but not the subsequent positive feedback events. As this component was observed in the first signal to change set (switch cue) and the first signal confirming an appropriate change in set (first positive feedback), this pattern was interpreted to confirm evidence of the role of the P300 in the reconfiguration of attention to changing task demands [16]. Given these findings in healthy adults, the absence or reduction of this expected marker of attention switching in set-shifting tasks may indicate a deficit in the processes that lead to efficient shifting behavior.

The role of feedback processing during the WCST may be investigated through the feedback-related negativity (FRN) response. Originally identified by Miltner et al. [21], the FRN is an ERP signal that is elicited by the processing of feedback. The FRN is identified as a negative-going ERP, maximal at fronto-central electrodes, and peaking between 250 and 300 ms after the onset of the feedback stimulus [21,22]. Holroyd and Coles [23] posit that the FRN is generated in the anterior cingulate cortex (ACC) and is linked to the executive control system, such that the FRN relates to the adaptive modification of behavior. The amplitude of the FRN is sensitive to the valence of the feedback (i.e., larger in response to negative feedback) and may depend on the extent to which feedback is informative and useful in improving performance—with larger FRNs suggesting greater utility of feedback to the task at hand [22]. In the WCST, shift feedback, or the first negative feedback in a set, serves primarily as a cue to switch but can also be processed as performance feedback. Evaluating the Cue P3 and FRN in relation to the shift feedback can shed light on the processing associated with the need to shift in children with and without DLD. 

#### 1.1.4. WCST in Children with DLD

Motivated by inconsistent findings regarding the performance of both children and adults with dyslexia on the WCST, Horowitz-Kraus [24] and Kraus and Horowitz-Kraus [25] compared electroencephalogram (EEG) components elicited during the completion of the WCST in adolescents with and without dyslexia. In their studies, individuals with dyslexia were found to commit more errors in the last trials of a set and have slower reaction times than peers with typical reading abilities. However, the ERP data did not reveal clearly differentiated patterns. In the first study, the electrophysiological responses to both the presentation of stimulus cards and the presentation of feedback signals were examined. Following the model put forth by Barceló [19], the study examined two feedback-locked components: the P3a as an indication of the activation of task-set information in working memory and the P3b as a reflection of the updating of task-set context in working memory. The results indicated that participants with dyslexia followed the same pattern as typical readers, with both groups showing larger P3a and P3b components in response to shift feedback as compared to stay feedback [24]. The study proposed that the lower behavioral performance of individuals with dyslexia was not related to shifting ability in and of itself but rather deficits in underlying attention or working memory abilities. The second study aimed to examine changes in cognitive processes during the WCST among individuals with and without dyslexia. The authors reported a pattern of a more pronounced increase in FRN amplitudes and a decrease in the P300s from early to late trials for individuals in the dyslexic group. They interpreted these findings to reflect the benefit of prolonged exposure to task learning for individuals with dyslexia [25]. 

### 1.2. Research Aims and Questions

Given the executive functioning skills of set-shifting and feedback processing central to the WCST, a comparison of the performance of typically developing children and children with DLD has the potential to offer insight into the relative status of these skills in children with DLD. Thus, the first aim of this study was to examine whether performance on a computerized version of the WCST by children with DLD and their age-matched, typically developing (TD) peers can be better understood by expanding on measures of accuracy to investigate patterns of performance in shifting, set-maintenance, and feedback processing. The second aim of this study was to determine whether children with DLD demonstrate differences in processing at the electrophysiological level compared to TD peers that may elucidate impairments in specific components of executive function related to the set-shifting and feedback processing. The ERP components of feedback processing (FRN) and attention shifting (P300), as elicited by the first negative (shift feedback), first positive (first stay), and last positive feedback events, were analyzed to determine the extent to which group differences exist and whether these reflect differences in cognitive processes associated with shifting and feedback processing. 

## 2. Materials and Methods

Data for this study were collected as a part of a larger ongoing study investigating the impaired learning mechanism in children with DLD. Participants included children between the ages of 8 years, 1 month and 10 years, 10 months who were recruited from the Greater Boston area, with English as a predominant language and normal or corrected hearing and vision. These children additionally had no diagnosis of autism, ADHD, or other known neurological deficits, no history of psychological or emotional disorders, nor a history of concussions/brain injury. All children achieved a standard score above the range of intellectual disability (i.e., SS > 80) on the non-verbal portion of the Kaufman Brief Intelligence Test, 2nd edition (KBIT-2). Children in the TD group had typical language development as indicated by a parental report and a raw Identification Core Score of 34 or greater on the Test of Integrated Language and Literacy Skills (TILLS). Children in the DLD group had impaired language as indicated by a parental report of language delay and a raw Identification Core Score of less than 34 on the TILLS. Participants completed an initial evaluation session, including assessments of language and cognitive abilities, to determine study eligibility and group membership. Data from 12 children with DLD and 12 typically developing (TD) age-matched peers were analyzed in this study. 

A summary of participant demographic and standardized assessment data is reported in Table 1. Comparison of group means indicated no significant difference in age (*p* = 0.67). The TD group achieved a significantly higher TILLS core language standard score than the DLD group, *F*(1,22) = 71.17, *p* < 0.001. There was also a significant difference between the group average KBIT-2 standard scores, *F*(1,22) = 6.03, *p* = 0.02. The TD group achieved a higher score than the DLD group; however, both were within the average range and well above the inclusion cut score. Additional measures of working memory and word reading fluency were administered to further describe the cognitive-linguistic profiles of the participants. There were significant group differences favoring those with TD on these descriptive assessments.

### 2.1. Task Procedure

**Figure 2:**
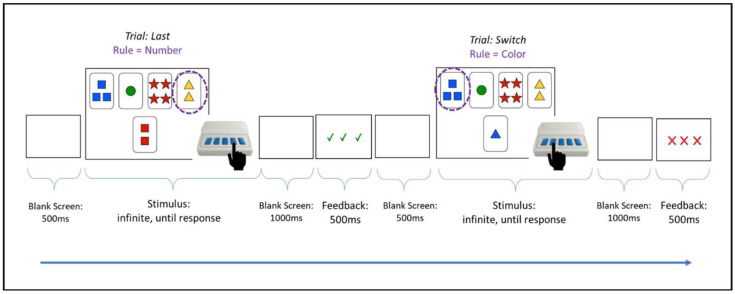
Timeline of presentation of task stimuli and participant response. For illustration purposes here, the encircled choice card on each slide represents the correct answer according to the current set rule of the stimulus. The button being pointed to on the response box represents expected participant behavior given current knowledge about the rules.

A computerized version of the Wisconsin Card Sorting Test was administered to participants in a quiet room while seated at a comfortable distance from a computer monitor adjusted to an appropriate eye-level height. Participants were fitted with a 32-channel EEG net, which continuously recorded EEG while the task was completed. During the task, stimuli consisting of 4 fixed cards (“key cards”) above 1 of 24 different cards (each a “choice card”) were presented to participants on the computer screen. For each stimulus, participants were instructed to select one of the key cards in the top row that matched the bottom choice card based on one of three rules related to the card stimulus characteristics—color, shape, or number—by pressing the button on a response box that corresponded to that card (e.g., farthest left key card = farthest left button). These choice cards were unambiguous in that they matched a key card based on only one stimulus characteristic. Participants were first shown a blank fixation slide for 500 ms before being shown the card stimulus slide, which was displayed until a response was pressed. After the response, feedback was presented for 500 ms, with a slide displaying three green checkmarks following a correct response or three red X’s following an incorrect response. Figure 2 presents the task stimuli and timeline of presentation, with an example of a correct response followed by an incorrect response due to the unannounced change in the task set rule (i.e., from sort by number to sort by color). 

The task consists of four sections: an instruction block, a paper-based practice protocol, a computer-based practice block, and a test block. The first section is a computer-based instruction block, which informs the participant of the three sorting rule principles and provides six practice trials following each rule (18 total trials). Participants are then instructed that the sorting rule will sometimes change unexpectedly and that they will need to determine a new rule. Pilot testing of the task led to the creation of a paper-based practice protocol that immediately follows the computer-based instruction block. In this training section, the participant is asked to demonstrate responses for each rule based on a given stimulus card, then practice maintaining one rule for multiple different cards, and finally practice switching to a new rule based on new negative feedback, with coaching by the task administrator to ensure the task is understood. A computer-based practice block follows, in which the participant is presented with 30 trials, with a set of ten trials for each of the three rules. These first sections are for the purpose of familiarizing the participant with the rules and structure of the task, and responses from these sections were not included in data analysis. 

The final section is the testing block. This block consists of 21 sets, where a set is a group of trials following one sorting rule. Each set has between seven and fourteen trials, such that the rule change does not happen at predictable intervals. Participants need to switch rules a total of 19 times in the testing block of the task. A “Switch” trial is the first trial of a new set governed by a new rule—in other words, Switch trials are those trials on which the unexpected rule change takes place. On the Switch trial, a participant is expected to respond according to the rule of the previous set (since they have not been warned of the rule change) and, therefore, should always receive negative feedback after this trial. The Switch 2 trial of a set is the participant’s first chance to respond to the shift in sets (i.e., change in the rule), and an ideal participant will have a 50% chance of finding the new rule (the previous rule has been discarded based on elicited negative feedback, and the participant must choose between the two remaining rules). Therefore, by the third trial of a set, an ideal participant will have enough information to sort by the correct rule, having discarded the previous set’s rule and either confirmed or ruled out the rule used on the Switch 2 trial. The same choice card displayed on the Switch trial stimulus is shown again on the Switch 2 trial, with the goal of reducing the load on working memory by enabling the participant to test a new sorting rule without a change in the choice card. If the incorrect rule is used on the Switch 2 trial, the same choice card is repeated one final time, with this third trial being labeled Switch 3. However, if the correct rule is guessed on the Switch 2 trial, a new choice card stimulus is presented on the third trial. After the Switch 3 trial, a new choice card is displayed regardless of accuracy on Switch 3. Other than Switch 2 and Switch 3 trials, the choice cards are not repeated in sequential trials. Due to the variable nature of the number of Switch 2 and 3 trials depending on participant accuracy within the new task structure, the total number of trials for each participant varies, falling between 223–232 trials. Sets are organized semi-randomly, such that each set follows a different rule than the previous set. Within the testing block, there are two short breaks to support participant attention. The sets immediately following breaks were excluded from analyses; therefore, data from a total of 19 sets were analyzed.

### 2.2. Behavioral Data Analysis

Performance on the testing block of the WCST was examined through measures that aimed to evaluate set-shifting and set-maintenance abilities, as well as effective behavior in response to feedback. Accuracy is the overall accuracy of responses calculated based on the proportion of correct responses across all trials, excluding the Switch (where errors are always expected) and Switch 2 (where errors are expected 50% of the time) trials. Within each rule set of the task, the learning criterion was defined as three consecutive correct responses at any point within the set. Based on this criterion, the total number of Sets Learned and the average number of Trials to Learn Set was also determined. 

To evaluate behavior within the set-shifting stage of the task, the Correct Shifts measure was calculated based on the proportion of rule shifts in which the participant responded with the correct rule within the two trials following the Switch trial (i.e., a correct response on Switch 2 and/or Switch 3). A correct rule shift was only considered if the participant had established the learning criterion on the previous set. As noted above, an ideal participant should be able to utilize trial and error to identify the correct rule by the Switch 3 trial of a set. Therefore, a perfect participant should achieve at least one correct response within the two trials following the Switch trial in 100% of the rule switch opportunities. As such, a lower proportion of Correct Shifts may be suggestive of a deficiency at the set-shifting stage of the task. To further elucidate difficulties at this stage of the task, perseverative errors within the first two trials following the Switch trial were also examined. Perseverative Shift Errors are error trials in which the participant responds according to the rule of the previous set. These errors suggest difficulty disengaging with the previous rule set and shifting to a new rule. The proportion of Switch 2 and Switch 3 trials in which the participant made a perseverative shift error was calculated from the total Switch 2 and Switch 3 trials. Only trials following a learned set were included in the calculation of Perseverative Shift Errors.

As success on the WCST hinges on the ability to effectively interpret negative feedback, we attempted to examine feedback processing separately from the process of shifting between the task’s changing rule sets. Because we expected that children would perform more poorly than healthy adults on this task, given their immature EF abilities, we expected more errors to be committed outside of the Switch trials and, therefore, more instances of negative feedback. Behavior following all negative feedback events was examined according to two categories of response on the trial immediately following the feedback presentation. An Effective Negative Feedback Response is a negative feedback presentation followed by a change in response rule used on the next trial. Conversely, feedback is considered ineffective when the participant fails to change rules and responds according to the same rule as the previous trial. The proportion of Effective Negative Feedback Responses was calculated out of the total instances of negative feedback. A summary of all behavioral variables is presented in Table 2.

Comparisons of the performance of the TD and DLD groups were analyzed using a series of one-way ANOVAs of group means for each of the behavioral variables. Additionally, the relationship between measures of set-shifting and accuracy, as well as the relationship between feedback processing and accuracy were examined through correlation analysis between accuracy and the variables of correct shifts, perseverative shifts, and effective negative feedback instances. 

### 2.3. EEG Data Acquisition and Analysis

EEG data were collected using the Electrical Geodesics Inc. (EGI; Eugene, OR, USA) 32-channel HydroCel Geodesic sensor net, composed of Ag/AgCl electrodes attached to an elastic net following the international 10–20 system. EEG was continuously recorded at a 1000-Hz sampling rate using the vertex as the reference electrode. Impedances were kept below 50 kΩ, and signals were acquired across all electrodes. The presentation of stimuli was controlled by programmable experiment generation software, E-Prime. MATLAB (The MathWorks, Inc.; Natick, MA, USA) together with the open-source EEGLAB toolbox version 2022.0 [30] were used for EEG data processing and analysis. Data were resampled at a 250 Hz sampling rate and filtered using a bandpass filter (0.1–30 Hz). The processed data were time-locked to the presentation of the visual feedback and segmented into 1200 ms epochs, including a 200 ms baseline. Each epoch was visually inspected for artifacts, and noisy epochs were manually removed. Baseline correction was performed on the averaged data based on signal in the 200 ms preceding the feedback stimulus, after which data were referenced to the average reference. ICA analysis was then applied to data for each subject to detect and correct for eye blink and movement artifacts. Of note, EEG data from four participants were not available, resulting in data from a total of nine participants with DLD and eleven with TD ultimately included in the ERP analysis.

The epochs to be analyzed were labeled according to five conditions. (1) First Negative Feedback (First NegFb) epochs are those negative feedback presentations elicited by the Switch trial, where the participant first received an indication that the previously established set rule is no longer valid. Negative feedback presentations were excluded if the previous set was not learned or if the participant made errors on two or more of the trials immediately prior to the Switch trial. These exclusions were intended to ensure that First Negative Feedback epochs captured only those instances of negative feedback in which a participant receives novel negative feedback for responding according to an established rule, and therefore represents an initial cue to “shift” from a learned rule set. (2) First Positive Feedback (First PosFb) epochs are the first positive feedback presentation of a set that is followed by at least one additional correct trial. First Positive Feedback trials, therefore, are equivalent to a cue to “stay” with the current rule set and represent the first time a participant received feedback confirming that they have correctly selected a new set rule and demonstrated repetition of that new rule. (3) Last Positive Feedback (Last PosFb) epochs are positive feedback presentations occurring in the last or second-to-last trial of a set. 

The remaining two variables analyzed were time-locked to feedback events occurring at any point along the task set and were based on the behavior of the participant following the feedback, thus representing Effective versus Ineffective Feedback events. (4) Effective Negative Feedback epochs are instances of negative feedback followed by a change in the response rule. In other words, they represent an effective response to feedback through a change in behavior. (5) Ineffective Negative Feedback epochs are instances of negative feedback that are followed by a response using the same rule—they represent negative feedback that is followed by ineffective behavior. Overall, the TD group demonstrated a few instances of Ineffective Negative Feedback. Five of the twelve TD participants had no Ineffective Negative Feedback trials, and an additional four had fewer than ten trials in this condition. Therefore, the Effective and Ineffective Negative Feedback conditions were only compared for DLD participants. 

A temporal principal component analysis (TPCA) was conducted to reduce the temporal dimensionality of the data set for each electrode. For the First Negative condition, TPCA at the frontocentral electrode (FCz) resulted in a set of six factors, with the FRN response captured by temporal factor 5, peaking at approximately 300 ms. TPCA at the parietal electrode (Pz) yielded a set of seven factors. Temporal factor 2 with a maximal peak around 400 ms captured a posterior P3 (P3b) component. Additionally, TPCA of the First Positive and Last Positive conditions at the Pz electrode resulted in a set of seven factors, accounting for 91.3% of the total variance. The P3b component was analyzed within an early and late time window, with temporal factor 1, maximal around 300 ms, and temporal factor 2, maximal around 800 ms. For the Effective and Ineffective Negative Feedback conditions, TPCA yielded a set of 14 factors at the FCz electrode, accounting for 83.00% of the variance. Temporal factor 3, maximal at approximately 250–300 ms, capturing the FRN component. The factor scores served as amplitude measures and were subjected to statistical analyses using IBM SPSS Statistics. 

To examine the relationship between the processing of feedback cues as reflected by the ERP components and behavioral outcomes, regression analyses were performed. The factor scores of the components related to switch and stay cues were used as predictors (First Negative FRN response, First Negative P3b response, First Positive early P3b response, and First Positive late P3b response) in a set of models with the behavioral outcomes Accuracy, Correct Shifts, and Perseverative Shift Errors each as dependent variables. Additionally, a set of models using responses to positive feedback (First Positive P3b response and Last Positive P3b response) as predictors was used with Accuracy, Correct Shifts, and Perseverative Shift Errors as dependent variables.

## 3. Results

### 3.1. Behavioral Data

A series of one-way ANOVAs were conducted to examine group differences between TD participants and DLD participants on each of the behavioral performance measures. Group averages and results of the ANOVAs are presented in Table 3. 

The average overall Accuracy rate was significantly greater for the TD group (M = 0.85, SD = 0.12) compared to the DLD group (M = 0.65, SD = 0.13), F (1, 22) = 14.31, *p* = 0.001. Of note, DLD participants demonstrated considerable variability with a median Accuracy level of 65% (range: 47% to 85%), while TD participants skewed positively with a median Accuracy level of 89% (range: 52% to 93%). In line with Accuracy, successful learning of rule sets varied among DLD participants, with the total number of Sets Learned varying from 9 to 19 sets out of 19 total sets (M = 14.25, SD = 3.41). Comparatively, TD participants showed a stronger ability to learn sets overall (M = 17.75, SD = 1.96), with an observed statistically significant difference between the TD and DLD group means, F (1, 22) = 9.48, *p* = 0.005. However, there was no significant difference observed between the groups in the average Trials to Learn Set, F(1, 22) = 1.60, *p* = 0.22. Both groups required 4-5 trials, on average, to learn a set rule. 

The TD group achieved a higher proportion of Correct Shifts (M = 0.88, SD = 0.20) than the DLD group (M = 0.71, SD = 0.16), with a statistically significant difference found between the groups, F(1, 22) = 5.09, *p* = 0.03. Conversely, the DLD group demonstrated a larger proportion of Perseverative Shift Errors on switch trials (M = 0.21, SD = 0.12) than the TD group (M = 0.09, SD = 0.10). This difference was also statistically significant, F (1, 22) = 8.03, *p* = 0.01. Across groups, a strong positive correlation was found between the proportion of Correct Shifts and Accuracy, r = 0.81, *p* < 0.001, and a strong negative correlation was observed between the proportion of Perseverative Shift Errors and Accuracy, r = −0.86, *p* < 0.001

Regarding the Effective Negative Feedback Response measure, the TD group demonstrated an overall greater tendency to follow the presentation of negative feedback with a change in their behavior. The TD participants followed negative feedback with an effective response in an average of 89% of negative feedback instances (SD = 0.11) compared to 78% (SD = 0.08) among the DLD participants, F(1, 22) = 8.43, *p* = 0.008. Across groups, a strong positive correlation was also found between the proportion of Effective Negative Feedback Responses and Accuracy, r = 0.85, *p* < 0.001.

### 3.2. ERP Data

#### 3.2.1. First Negative Feedback

**Figure 3:**
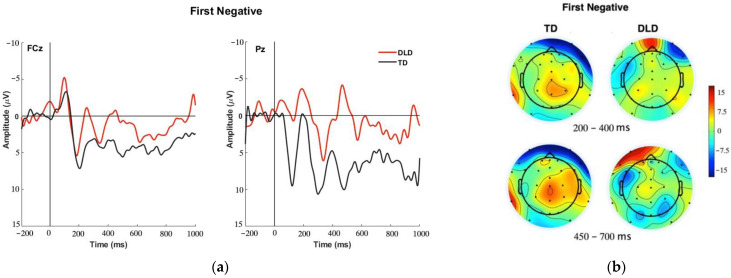
(**a**) Grand average event-related potential (ERP) waveform at the frontocentral electrode (FCz—**left**) and parietal electrode (Pz—**right**) for First Negative Feedback (FN), for participants with typical development (TD—black line) and participants with Development Language Disorder (DLD—red line). (**b**) Topographic maps in early and late time windows (200–400 ms and 450–700 ms) for First Negative in TD (**left column**) and DLD (**right column**) participants.

**Figure 4:**
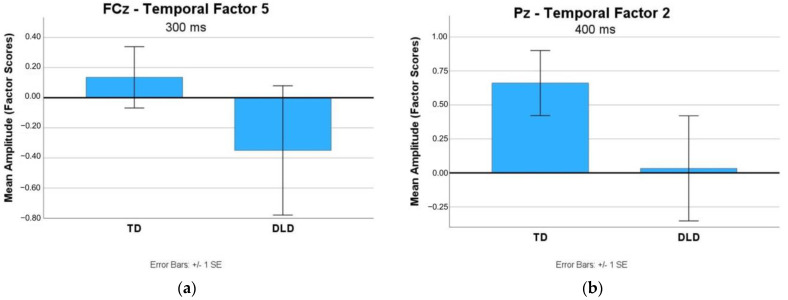
(**a**) Mean amplitudes (in factor scores) of the feedback-related negativity (FRN) component maximal at around 300 ms (temporal factor 5) yielded by Temporal Principal Component Analysis (TPCA) at FCz. (**b**) Mean amplitudes of the posterior late component maximal at around 400 ms (temporal factor 2) yielded by TPCA at Pz.

To examine the processing of the cue to shift between task sets, the ERPs time-locked to First Negative Feedback events were compared between the two groups. Figure 3 presents the topo-map of the First Negative Feedback and the grand average ERP waveforms associated with the feedback events captured at FCz and Pz in the two groups. Visual inspection suggests that while the first negative feedback elicited a parietal positivity in the TD group, it elicited a fronto-central FRN-like activity in the DLD group. We first examined FCz for the presence of the P3a and/or FRN components in relation to the first negative feedback. A P3a activity was not detected in either group. The FRN amplitudes (temporal factor 5 scores with a peak at 300 ms at FCz) were compared between the two groups. While group differences were not found significant, *t* (18) = 1.08, *p* = 0.14, a trend toward larger negativity in the DLD group emerged. Figure 4a presents a comparison of the mean amplitudes (factor scores) yielded by the TPCA within this time window. A posterior P3 component (P3b) was captured at the Pz electrode in the two groups in the time window between 300 and 500 ms (temporal factor 2). Statistical analysis did not yield a significant difference between the groups, *t* (18) = 1.43, *p* = 0.08. However, the participants with TD demonstrated a trend of a positive-going waveform consistent with a P3b in response to the Negative Feedback. A comparison of the mean amplitudes yielded by the TPCA at Pz is presented in Figure 4b.

#### 3.2.2. First Positive versus Last Positive Feedback

**Figure 5:**
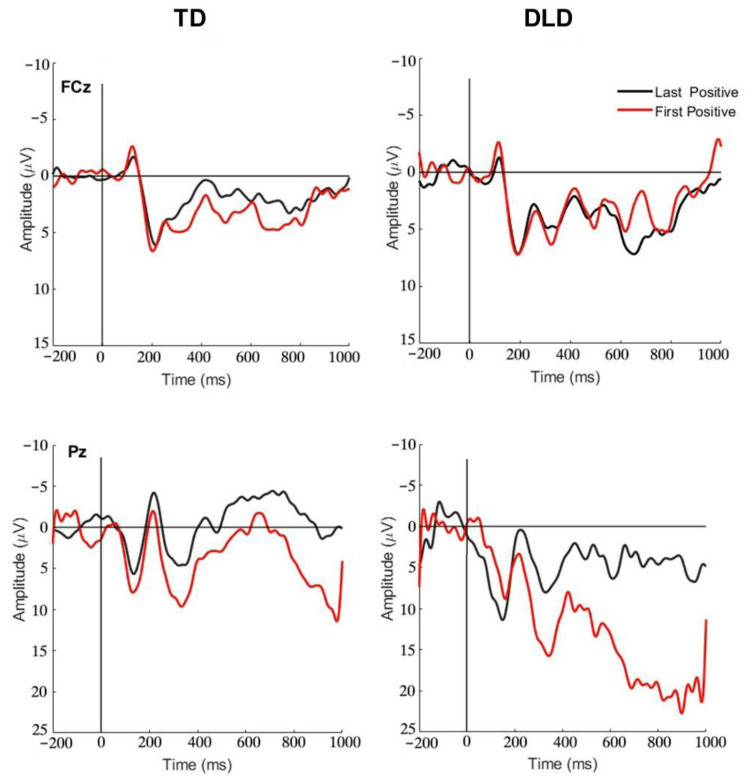
Grand average ERP waveform at the frontocentral electrode (FCz—**top row**) and parietal electrode (Pz—**bottom row**) for First Positive Feedback (red line) and Last Positive Feedback (black line) for participants with typical development (TD—**left column**) and participants with Development Language Disorder (DLD—**right column**).

**Figure 6:**
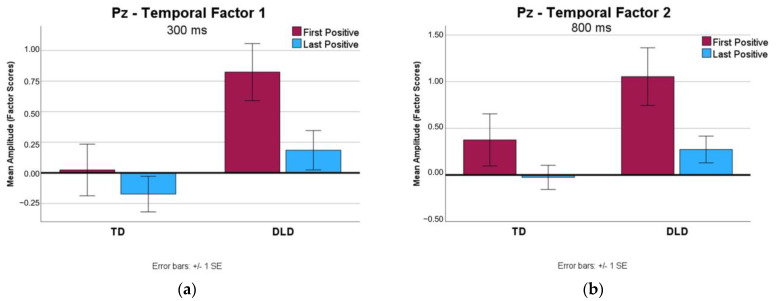
(**a**) Mean amplitudes (in factor scores) yielded by TPCA with a maximal peak around 300 ms (temporal factor 1) at Pz for First Positive and Last Positive Conditions. (**b**) Mean amplitudes yielded by TPCA with a maximal peak around 800 ms (temporal factor 2) at Pz, for First Positive and Last Positive Conditions.

The P3 component associated with the First Positive and Last Positive Feedback events was compared between the two groups. In Figure 5, the grand average ERP waveforms associated with the First Positive and Last Positive Feedback events captured at FCz and Pz in the two groups are presented. Electrode Pz captures clear differences between the two types of feedback events that are consistent with the P3b. A comparison of the mean amplitudes (factor scores) yielded by the TPCA within the early and late time windows that peaked at 300 ms (temporal factor 1) and 800 ms (temporal factor 2) is presented in Figure 6. An ANOVA comparing the Last Positive and First Positive conditions between the groups for the early peak (TF 1) resulted in a significant group effect, *F* (1, 18) = 7.70, *p* = 0.01, ŋ^2^ = 0.30, suggesting a larger positive amplitude in the DLD group. A significant condition effect was also found, *F* (1, 18) = 5.95, *p* = 0.03, ŋ^2^ = 0.25, indicating that a larger positivity was elicited by the First Positive feedback than by the Last Positive feedback. No significant interaction between group and condition was found, *F* (1, 18) = 1.66, *p* = 0.21, ŋ^2^ = 0.08. The analysis of the late positivity yielded similar patterns of results, with group and condition effects (Response: *F* (1, 18) = 6.82, *p* = 0.02, ŋ^2^ = 0.28; Group: *F* (1, 18) = 4.41, *p* = 0.05, ŋ^2^ = 0.19), but no interaction, *F* (1,18) = 0.70, *p* = 0.41, ŋ^2^ = 0.04.

#### 3.2.3. Effective versus Ineffective Feedback

**Figure 7:**
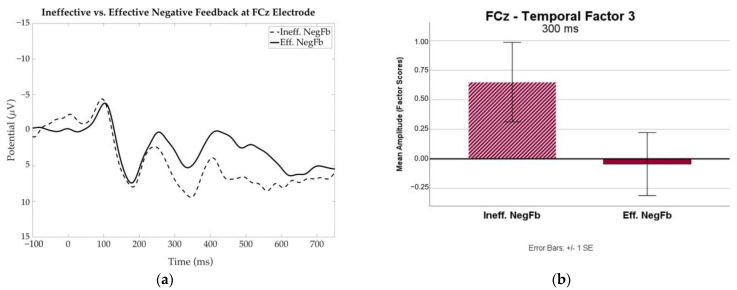
(**a**) Grand average ERP waveform at the frontocentral electrode (FCz) for Ineffective Negative Feedback (Ineff. NegFb) and Effective Negative Feedback (Eff. NegFb) conditions for participants with Development Language Disorder (DLD). (**b**) Mean amplitudes (in factor scores) of the FRN component maximal at around 300 ms (temporal factor 3) yielded by TPCA at FCz.

To examine differences in the neural activity associated with effective versus ineffective feedback events, the FRN related to these conditions was examined for the DLD participants. Temporal factor 3, with a maximal peak around 250–300 ms, was associated with the FRN at the FCz electrode. Figure 7 presents the grand average ERP waveforms associated with the Effective Negative Feedback (Eff. NegFb) and Ineffective Negative Feedback (Ineff. NegFb) events at FCz. An independent samples *t*-test of the mean amplitudes of the factor scores revealed a difference in the Effective versus Ineffective Feedback conditions that approached significance, *t* (12) = 1.62, *p* = 0.06. The mean amplitude was larger (more negative) for the Effective Negative Feedback condition, as reflected by the more negative-going peak seen in the waveform for this condition at approximately 250 ms.

### 3.3. Regression Analyses

The results of the regression analyses that evaluated the contribution of ERPs elicited by First and Last Positive Feedback events to Accuracy, Correct Shifts, and Perseverative Shift Error outcomes for the two participant groups are presented in Table 4. For children with TD, this model indicated a significant negative contribution of the early P3b elicited by First Positive Feedback to measures of Correct Shifts (*β* = −0.703, *p* = 0.023) and a positive contribution to measures of Perseverative Shift Errors (*β* = 0.886, *p* = 0.014). An opposite effect was found for the P3b elicited by Last Positive Feedback, with a significant positive contribution to Correct Shifts (*β* = 1.070, *p* = 0.003) and a negative contribution to Perseverative Shift Errors (*β* = −0.934, *p* = 0.010). These results indicate that, for TD participants, better performance on shift trials was associated with smaller P3b responses to the First Positive Feedback events but larger P3b responses to the Last Positive Feedback events. These ERP components were not found to be significant predictors of the overall Accuracy measure for TD participants. For participants with DLD, the only behavioral measure found to be significantly predicted by these components was Perseverative Shift Errors. More specifically, there was a significant contribution of the P3b elicited by the Last Positive Feedback to Perseverative Shift Errors. Contrary to the findings for TD participants, for participants in the DLD group, a larger Last Positive P3b response was associated with a greater proportion of Perseverative Shift Errors. The contribution of the FRN elicited by First Negative Feedback to Correct Shifts was not found to be significant; however, a trend emerged suggesting that a smaller FRN (greater positive amplitude) was associated with better Correct Shift outcomes (*β* = 0.688, *p* = 0.068) in the TD group.

## 4. Discussion

### 4.1. Behavioral Outcomes of TD and DLD Participants

Across several behavioral measures used to examine performance on the WCST in this study, participants with DLD demonstrated poorer results compared to their peers with TD. On typical measures of successful WCST completion, accuracy, and number of sets learned, the DLD group had lower outcomes, suggesting overall greater difficulty completing this task. This finding is in line with previous studies that have demonstrated a disadvantage among children with DLD in terms of accuracy measures on the WCST [9,10]. However, given the complexity of this task, this measure alone does not provide sufficient insight into what aspects of the task may have caused it to be more challenging for children with DLD to successfully complete. 

The measures of Correct Shifts and Perseverative Shift Errors were examined to evaluate participants’ responses to cues to switch sets and their adjustments to new rules. On both measures, participants with DLD demonstrated impaired performance compared to their TD peers, highlighting greater difficulty disengaging from the previous set rule as well as quickly identifying a new rule. Participants with DLD had more errors on switch trials than their TD peers, and they were more likely than their peers to continue responding based on a previous rule after the set shift. However, many switch errors committed by children with DLD were random rather than perseverative. This observation suggests that the difficulty establishing a new rule may not be isolated to shifting away from a previously established rule but may be more related to difficulty establishing a rule to begin with, coupled with limited working memory capacity and inefficient use of feedback to identify the need to change a behavior. Based on their finding of similar rates of perseverative and non-perseverative errors in the early trials of WCST sets among patients with prefrontal lesions, Barceló and Knight [15] suggested these patients exhibited difficulty stemming not merely from being “stuck-in-set” but also from deficiency attending to a newly relevant category in the presence of distracting stimulus features. Given the evidence that children with DLD demonstrate deficits in interference control [4,5], similar difficulties specifying attention to a single target feature, among other possible matches, may have impacted performance. Thus, participants in the present study with a relatively small proportion of perseverative shifts, fewer correct shifts, and lower overall accuracy may exhibit impaired shifting that stems from difficulty establishing a task set rather than from disengaging a previously learned one. 

One measure that was not found to be significantly different between the groups was the average number of trials to learn a set. Despite demonstrating a low proportion of correct rule shifts, children with DLD established the learning criterion for a set (three consecutive correct responses) in an average of just under five trials. These two apparently opposing trends can be understood in the context of fewer total sets learned by the DLD group. They may also suggest that not establishing a rule within the first few trials in a set was detrimental to the success of children with DLD in establishing a rule within the set. In other words, children with DLD were less effective at recovering from errors in shift trials to then adapt their behavior and determine the new set rule.

When considering responses to negative feedback throughout the task, the DLD group demonstrated lower proportions of effective responses (i.e., switch behavior) compared to the TD group, suggestive of a weakness in immediately adapting upon receipt of negative feedback. Success on the WCST task relies on the ability to interpret negative feedback to appropriately reject an incorrect rule and then adjust behavior on the following trial. This is key not only at the set-shifting stage of the task but also to “recover” from the set-loss errors made at any point along the task. It is not uncommon for children to have difficulty using negative feedback to adjust their behavior. For example, when provided with corrective feedback during a probabilistic classification task, both children with DLD and TD used negative feedback to change their responses just 50% of the time. They were only slightly better at using positive feedback to maintain correct responses (on 52% of opportunities), a pattern of performance that differed from TD peers, who used positive feedback to maintain correct responses on 63% of opportunities [31]. In the present study, “switch” behavior, or an effective response to negative feedback, was considered as any change in behavior, correct or incorrect, following negative feedback. Given the three possible sorting rules, there was a two-in-three chance of responding with a “switch” after negative feedback. The significant difference between the groups related to effective responses to negative feedback highlights the difficulty among children with DLD to use negative feedback to change their behavior compared to TD peers, notwithstanding an ability (or lack thereof) to recall or narrow down the correct rule. These results align with evidence that children with DLD demonstrate inefficient feedback processing [18,31]. Further analysis of the responses to positive feedback in this task—e.g., an examination of “set loss” errors, where participants commit a change in response rule despite positive feedback—may help to expand on this understanding of the relative status of feedback processing abilities in the two groups of participants. 

Of note, large standard deviations were observed across behavioral measures for participants with DLD, suggesting considerable diversity in performance within this group. The differing levels of performance observed among children with DLD in our sample are reflective of the variability seen in previous studies examining executive functions in this population [3,4]. Reporting on large standard deviations observed in the accuracy measures found in a study of WCST performance among both individuals with dyslexia and TD peers, Horowitz-Kraus [24] suggested that the unequal development of executive functions may have contributed to the diverse results in the study’s sample of adolescents. In a cross-sectional study comparing WCST performance by typically developing children from four different age groups, Huizinga and van der Molen [32] found improvements in the proportion of correct responses from 7-year-olds to 11-year-olds and from 11-year-olds to 15-year-olds. Given that the participants in the current study encompass an age range across which improvement in performance on the WCST has been identified, this sample may have been more likely to capture a wide range of abilities stemming from this period of development. A follow-up analysis of the current results may help elucidate whether the variability in performance may be related to variability in participant age or whether other underlying differences impacted these results. 

### 4.2. ERP Responses

For participants with TD, ERPs time-locked to the First Negative Feedback condition revealed patterns reflective of a Cue P3 response at the parietal electrode. This positive-going ERP response was absent for the participants with DLD. In contrast, children in the DLD group showed an FRN-like activity in response to the first negative feedback. The pattern of ERP responses demonstrated within the TD group aligns with the P3b responses elicited by cues to shift rule sets within the WCST demonstrated in previous studies with adult subjects [13,15] as well as in a study of adolescents [24]. To the extent that the P3b reflects context-updating, the absence of this response among participants with DLD is suggestive of less efficient use of this negative feedback cue to update their task-set information in working memory. A P3b in response to negative feedback has also been interpreted as a marker of feedback informativeness, as opposed to valence [33]. The lack of a reliable P3b to negative feedback among children with DLD is further evidence that they are less attuned to the informative value of negative feedback. It is also in alignment with behavioral data showing that children with DLD have difficulty using negative feedback effectively or in the context of the WCST task, interpreting feedback as an informative switch cue. The trend of larger amplitude P3b response among participants with TD compared to participants with DLD suggests a potential weakness among the latter group in the processing that leads to successful updating of the rules after a signal to change. Given that these participants made more errors and learned fewer sets, the absence of a P3b response in the DLD group may further be suggestive of a weakened representation of the task set rules in working memory.

While the DLD group may not have demonstrated processing of the First Negative Feedback as a cue to shift sets, trends in the waveform do suggest an FRN response was elicited, indicative of processing of the cue as typical negative feedback. This may suggest that the children with DLD in our sample tended to process this negative feedback as an indication that their behavior was incorrect. However, such an indication was not translated into a cue that leads to an action. These ERP trends align with the behavioral outcomes demonstrating the overall difficulty of the participants with DLD to make a correct response at the beginning of a set, as well as to successfully learn set rules within the task. 

Interestingly, for participants with TD, the amplitude of the FRN component elicited by the First Negative Feedback demonstrated a trend toward predicting Correct Shifts such that the smaller the FRN (more positive), the better the performance (more Correct Shifts). Previous studies have reported similar findings of a smaller FRN associated with better performance and learning on tasks involving feedback [34]. The authors interpreted these findings to suggest that a smaller FRN is reflective of more efficient processing of negative feedback, with fewer cognitive resources being used to extract information from feedback [34]. Conversely, the larger overall FRN exhibited among participants with DLD in the present study suggests more significant resources allocated to the processing of negative feedback as a result of less developed feedback processing abilities. For participants with DLD, there was no relationship found between negative feedback processing as measured by the FRN and performance.

To the extent that DLD and dyslexia are both language-based learning disorders, it is interesting that our findings do not align with that of Horowitz-Kraus [24], who found that adolescents with dyslexia demonstrated a P3b response to switch cues or negative feedback. Notably, Horowitz-Kraus did not find significant differences in error rates in early trials of the set between children with and without dyslexia, in contrast to deficits that were observed in the DLD group of this study. The contrast in behavioral outcomes and electrophysiological patterns by the previous study’s participants with dyslexia and the participants with DLD in the current study may be a result of a difference in the age of the groups (i.e., a mean age of 9.44 years for DLD participants in the current study, compared to 12.85 years in the dyslexia study), with older participants having better developed EF skills and processing abilities. Additionally, such differences may point to differences in language-based skills that differentiate the disorders of DLD and dyslexia, and that may support performance on the WCST.

A comparison of ERPs elicited by the First Positive and Last Positive Feedback events demonstrated a trend of a strong P3b response to the First Positive that was diminished in response to the Last Positive. Arbel et al. [22] reported an association of the P3b to positive feedback to learning across a paired-associates task, interpreted to reflect the relevance of positive feedback in the learning process, with larger P3b amplitudes reflecting less expected feedback. The response to the First Positive Feedback is indicative of the ability of both groups to effectively use positive feedback to guide behavior after the change in sets. Additionally, the reduction in response from the First to the Last Positive Feedback events suggests that both groups learned across the task set and became more confident with their responses. As learning progressed within a set, they were less reliant on positive feedback to confirm accuracy. These trends are in line with previous studies that demonstrated a reduction in the P3b elicited in response to second “stay” cues (i.e., cues that provide redundant confirmation of a current task set) or later-occurring positive feedback events as compared to the P3b elicited by the switch cue [15,24]. Given the evidence that the P3b response is related to the informative value or the utility of positive feedback [33,35], the trend of reduced P3b responses to the Last Positive Feedback events suggests that participants demonstrated learning of the set rule across the task that diminished the salience of the information provided by these feedback events. The group differences that emerged pointing to a smaller P3b in response to positive feedback among participants with TD may suggest that positive feedback was less unexpected or less relevant for these participants. One explanation may be that participants with TD were able to gain more information (i.e., narrow down the new rule) using the earlier-occurring negative feedback, making the positive feedback less informative. Our data suggest that this was not the case for participants with DLD.

The results of the regression analyses suggested that, for participants with DLD, larger amplitudes of the P3b elicited by the Last Positive Feedback events were associated with more Perseverative Shift Errors. This finding aligns with expectations, given the interpretation that a smaller P3b in response to the Last Positive Feedback indicates better learning. Thus, DLD participants who were better able to learn a set rule (and thus were less reliant on positive feedback events occurring late in the set) were then less likely to make perseverative errors after a change in sets. 

The relationship between the P3b to positive feedback and behavioral outcomes for participants with TD was found to be distinct from that observed for participants with DLD. Regression analyses suggested that, in the TD group, a larger P3b amplitude elicited by the Last Positive Feedback contributed to more Correct Shifts and fewer Perseverative Shift Errors (i.e., better performance). By contrast, this same pattern of behavioral outcomes was predicted by a smaller P3b amplitude elicited by the First Positive Feedback. This suggests that less attentional resources devoted to the First Positive Feedback and more attention to the Last Positive Feedback were indicative of better processing among TD participants. These results were not expected, given evidence that a reduction in the P3b elicited by consecutive positive feedback events was associated with learning across a feedback-based task [22]. Further, larger ERPs related to the first positive feedback, as opposed to later occurring feedback, have been found to be most strongly associated with learning outcomes [35]. The structure of the set-shifting in the WCST task may offer a potential explanation for the different patterns observed in this study. As noted above, a smaller P3b amplitude in response to the First Positive Feedback may indicate that this “stay” cue was less novel and less informative for participants who were able to update the representation of the set rule based on the preceding negative feedback, which reduced the uncertainty about the next sorting rule. The association between a larger P3b to the Last Positive Feedback and better performance following the shift may have reflected anticipation of a shift. More specifically, participants who had a strong understanding of the task may have been able to anticipate the shift in sets to some extent, making positive feedback events in the later trials in a set important to attend to. Although the variable number of trials in a set was intended to prevent the anticipation of the rule switch, it is possible that after a certain number of correct trials, participants began to expect negative feedback signaling the change in sets. Perhaps this uncertainty toward the end of a learned set among participants with a heightened sensitivity to the potential set shift impacted the processing of the Last Positive Feedback event.

The presence of a larger P3b response to the First Positive Feedback event by children with DLD coupled with the lack of P3b in response to the First Negative Feedback may suggest that children with DLD rely more strongly on positive feedback than negative feedback to guide performance and learning. Within the WCST, negative feedback requires participants to hold the current response in working memory while selecting one of two competing hypotheses to test on the next trial. Positive feedback, on the other hand, indicates to the participant that the current rule they selected is correct. While working memory plays a role in maintaining the rule, the load on the limited resources of attention and memory is much heavier when one needs to establish a rule in response to a cue to switch. Using evidence from a paired-associates learning task, previous studies have suggested that there is a stronger association between learning outcomes and the processing of positive feedback, as opposed to negative feedback [22,35]. One proposed interpretation is that the greater demands of processing negative feedback make it more difficult for individuals to learn from it [35]. For participants with DLD in particular, who are purported to have deficits in attention and memory, this explanation of reliance on positive feedback for learning appears to hold true within the context of the WCST. 

The trend toward more robust FRN for the effective feedback condition among participants with DLD suggests that effective behavioral responses to feedback may be reflected in the processing of feedback at the electrophysiological level. When children with DLD responded with a change in behavior, they exhibited a stronger response to negative feedback. Such differences in processing may suggest that the failure of these participants to correct errors within this task may be a result of reduced attention to or inefficient processing of negative feedback as a signal to change behavior. The FRN patterns suggest that participants with DLD may exhibit weakness in this initial processing of the feedback cue. Given the role of feedback in the WCST, such a processing deficit is likely to impact overall performance in both correcting random errors as well as shifting between sets.

For children with TD, the absence of a distinctive frontocentral P3a, and the presence of a more posterior P3b pattern in response to the cue to shift point to possible processing or strategy use differences at the set-shifting stage of the task for the young participants in this study as compared to adults [16,19] or even slightly older children [24]. Cunillera et al. [16] cite evidence of widely distributed brain network activation in response to the need to change task sets within the context of the WCST. They suggest that both cues to shift sets and cues to maintain a newly established set engage analogous cognitive control mechanisms that require the coordination of several brain regions [16]. It warrants further investigation whether the sample of children in this study demonstrates different patterns in P300 responses as a result of developmental differences in the differentiation and/or coordination of these cognitive control mechanisms. 

### 4.3. Limitations and Future Directions

A few limitations of this study must be noted. Overall, the small number of participants has an impact on the extent to which these results may be representative of the population and indicative of true trends. Further, the heterogeneity in presentation of DLD among children, such as varying severity of language impairments, has been noted to influence outcomes in measures of executive function [5]. However, despite the small sample, analyses of behavioral outcomes resulted in overall large effect sizes, with η^2^ ranging from 0.068 to 0.394, suggesting a clear distinction between the two groups of participants. It is likely that the factors that limited the sample size, including the careful matching of the two groups on age and gender and the exclusion of participants with comorbid conditions such as attention deficit disorder, contributed to our ability to observe large effect sizes. Due to performance patterns on the task, there was a variable number of trials included for each of the conditions across participants, and the overall limited number of trials captured in the time-locked data was then further reduced once noisy artifacts were removed. The relatively small number of trials included for analysis for each participant may detract from the robustness of ERP components and the statistical analyses.

The findings of this study offer insight into patterns of performance on the WCST that may serve as a departure point for future investigation into executive functioning in children with DLD and their TD peers. Given the variability observed in the performance of DLD participants, further analyses of different profiles of performers may shed light on different strategies employed in the task, as well as suggest different sources of difficulty. One possibility may be to expand upon a comparison of WCST performance and other measures of executive functioning, particularly other measures that capture shifting ability. Additionally, the investigation of ERPs time-locked to different task events may expand on the trends observed in the present variables. For example, an examination of positive feedback followed by an incorrect response could expand upon understanding of the role of feedback in this task. Other studies (e.g., Barcelo et al. [15], Horowitz-Kraus [24]) have examined ERPs linked to target card presentation. In light of THE proposed feedback processing deficits among the DLD group, the stimulus card processing stage of the task may highlight another source of differences between participants who effectively versus ineffectively completed this task.

## 5. Conclusions

This study provided an in-depth behavioral and electrophysiological examination of set-shifting and feedback-processing of children with DLD compared to their TD peers based on performance on a computerized Wisconsin Card Sorting Test (WCST). Behaviorally, children in the DLD group demonstrated poorer outcomes on nearly all measures examined when compared with their peers. Behavioral measures highlighted the challenges participants with DLD experienced relative to TD peers in effectively using feedback to learn through trial and error, as well as flexibly shifting between sorting rules. The ERP results pointed to inefficient processing of negative feedback by children with DLD in the context of rule-shifting. The findings of this study lend support to existing evidence that children with DLD demonstrate deficits in non-verbal tasks of executive function, and with feedback-based learning in particular. Though the WCST is typically considered to be strongly associated with the executive function of shifting, it appears that difficulties with the processing of negative feedback contributed to the poor performance of children with DLD in our sample. Given the relative disadvantage of children with DLD in this study to use negative feedback effectively to inform behavior, it is important to consider how feedback is implemented within instruction and intervention for this population. Further, electrophysiological evidence for more robust use of positive feedback to update task-set information suggests there may be greater value in structuring interventions that make use of positive, confirmatory feedback for children with DLD. While evidence from this study suggests that children with DLD encounter challenges when the need to shift behavior is presented through negative feedback, future work should aim to shed light on the impact of shifting on learning and performance when rule shifts are more explicit. 

## Figures and Tables

**Table 1:** Participant demographics and standardized assessment scores for Typically Developing (TD) and Developmental Language Disorder (DLD) participants.

	TD (*n* = 12)	DLD (*n* = 12)	One-Way ANOVA			
			F	df	*p*	η^2^
Age (months)	115.47 (12.66)	113.32 (11.51)	0.19	22	0.67	0.009
TILLS Identification Core Standard Score	106.83 (11.73)	68.75 (10.34)	71.17	22	<0.001	0.764
KBIT-2 Nonverbal Standard Score	109.75 (13.90)	96.91 (11.60)	6.03	22	0.02	0.215
CELF-4 Working Memory Index Score	102.33 (11.40)	89.83 (12.31)	6.66	22	0.02	0.232
TOWRE-2 Total Standard Score	109.00 (9.98)	77.50 (17.12)	30.31	22	<0.001	0.579
			Chi-Square Test			
			χ^2^	df	p	
Gender	6 Male 6 Female	6 Male 6 Female	0.00	1	1.00	

Note: TILLS = Test of Integrated Language and Literacy Skills [26]; KBIT-2 = Kaufman Brief Intelligence Test, 2nd Edition [27]; CELF-4 = Clinical Evaluation of Language Fundamentals, 4th Edition [28]; TOWRE-2 = Test of Word Reading Efficiency, 2nd Edition [29].

**Table 2:** WCST behavioral measures and definitions.

Measure	Definition
Accuracy	The proportion correct across all trials, excluding Switch and Switch 2 trials.
Sets Learned	The number of sets where participant responded with the correct rule on at least three consecutive trials (regardless of any other errors in this set) out of 19 total sets.
Trials to Learn Set	The average number of trials on which a participant established “learning criterion” (three consecutive correct responses) across all learned sets. Excludes Switch trial so that trial 1 of a set = Switch 2 trial.
Correct Shifts	The proportion of rule shifts where the participant finds the correct rule on either the Switch 2 and/or Switch 3 trial out of total rule shifts. Only includes rule shifts where the participant successfully learned the previous set and the participant did not “anticipate” the rule shift.
Perseverative Shift Errors	The proportion of rule shifts where the participant’s response on the Switch 2 and/or Switch 3 trials is made according to the rule of the previous set out of total Switch 2 and Switch 3 trials. Only rule shifts where the participant successfully learned the previous set.
Effective Negative Feedback Response	The number of negative feedback presentations that are followed by a *change* in the participant’s response (i.e., participant does not respond according to same rule that elicited negative feedback) as a proportion of the total instances of negative feedback. Excludes feedback events where the subsequent response is a “No Rule” response (i.e., button press does not map onto any of the rules).

**Table 3:** Behavioral measures of WCST performance.

Measure	TD (*n* = 12)	DLD (*n* = 12)	One-Way ANOVA			
			F	df	*p*	η^2^
Accuracy	0.85 (0.12)	0.65 (0.13)	14.31	22	0.001	0.394
Sets Learned	17.75 (1.96)	14.25 (3.41)	9.48	22	0.005	0.301
Trials to Learn Set	4.43 (0.68)	4.89 (1.06)	1.60	22	0.22	0.068
Correct Shifts	0.88 (0.20)	0.71 (0.16)	5.09	22	0.03	0.188
Perseverative Shift Errors	0.09 (0.10)	0.21 (0.11)	8.03	22	0.01	0.267
Effective Negative Feedback Response	0.89 (0.11)	0.78 (0.08)	8.43	22	0.008	0.277

**Table 4:** Results of regression models using factor score measures of P3b (temporal factor 1) to First Positive and Last Positive Feedback and FRN to First Negative Feedback to predict Accuracy, Correct Shifts, and Perseverative Shift Errors.

		TD			DLD		
Model		Beta	t	Sig.	Beta	t	Sig.
Accuracy	First Positive P3b	−0.601	−1.754	0.117	−0.075	−0.197	0.851
	Last Positive P3b	0.840	2.452	0.040	−0.432	−1.127	0.303
	F	3.045		0.104	0.639		0.560
	Adj. R2	0.290			−0.099		
Correct Shifts	First Positive P3b	−0.703	−2.793	0.023	0.560	1.724	0.135
	Last Positive P3b	1.070	4.249	0.003	−0.198	−0.611	0.563
	First Negative FRN *	0.688	2.225	0.068	0.425	1.155	0.312
	F	9.039		0.009	2.072		0.207
	Adj. R2	0.617			0.211		
Perseverative Shift Errors	First Positive P3b	0.886	3.153	0.014	0.226	1.391	0.214
	Last Positive P3b	−0.934	−3.323	0.010	0.954	5.868	0.001
	F	6.458		0.021	17.223		0.003
	Adj. R2	0.522			0.802		

* Note: The analysis of FRN as a predictor of Correct Shifts resulted from a regression model using FRN and P3b elicited by the First Negative Feedback and P3b elicited First Positive Feedback as predictors. This model was only found to significantly predict Correct Shifts for the TD participants (Adj. R^2^ = 0.609, *F* = 4.896, *p* = 0.043).

## Data Availability

The data presented in the current study are available from the corresponding author upon reasonable request.
